# HRD1 inhibits fatty acid oxidation and tumorigenesis by ubiquitinating CPT2 in triple‐negative breast cancer

**DOI:** 10.1002/1878-0261.12856

**Published:** 2020-12-16

**Authors:** Xin Guo, Aman Wang, Wen Wang, Ya Wang, Huan Chen, Xiaolong Liu, Tian Xia, Aijia Zhang, Di Chen, Huan Qi, Ting Ling, Hai‐long Piao, Hong‐jiang Wang

**Affiliations:** ^1^ First Affiliated Hospital of Dalian Medical University Dalian Medical University China; ^2^ CAS Key Laboratory of Separation Science for Analytical Chemistry Dalian Institute of Chemical Physics Chinese Academy of Sciences Dalian China; ^3^ Liaoning Key Laboratory of Molecular Targeted Drugs in Hepatobiliary and Pancreatic Cancer Dalian China; ^4^ University of Chinese Academy of Sciences Beijing China; ^5^ Department of Biochemistry & Molecular Biology School of Life Sciences China Medical University Shenyang China

**Keywords:** CPT2, FAO, HRD1, TNBC, ubiquitination

## Abstract

Dependence on glutamine and acceleration of fatty acid oxidation (FAO) are both metabolic characteristics of triple‐negative breast cancer (TNBC). With the rapid growth of tumors, accelerated glutamine catabolism depletes local glutamine, resulting in glutamine deficiency. Studies have shown that the use of alternative energy sources, such as fatty acids, enables tumor cells to continue to proliferate rapidly in a glutamine‐deficient microenvironment. However, the detailed mechanisms behind this metabolic change are still unclear. Herein, we identified HRD1 as a regulatory protein for FAO that specifically inhibits TNBC cell proliferation under glutamine‐deficient conditions. Furthermore, we observed that HRD1 expression is significantly downregulated under glutamine deprivation and HRD1 directly ubiquitinates and stabilizes CPT2 through K48‐linked ubiquitination. In addition, the inhibition of CPT2 expression dramatically suppresses TNBC cell proliferation mediated by HRD1 knockdown *in vitro* and *in vivo*. Finally, we found that the glutaminase inhibitor CB839 significantly inhibited TNBC cell tumor growth, but not in the HRD1 knock‐downed TNBC cells. These findings provide an invaluable insight into HRD1 as a regulator of lipid metabolism and have important implications for TNBC therapeutic targeting.

## Graphical abstract

In the current study, we demonstrated that HRD1 was downregulated in TNBC tissues and dysregulated FAO by ubiquitinating CPT2, especially under glutamine‐deficient conditions. In addition, the curative effect of CB839 is more prominent in TNBC cells and tumors with high HRD1 expression. These findings provide insight into HRD1 as a regulator of lipid metabolism and have important implications for TNBC therapeutic targeting.

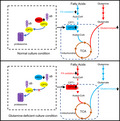

**Abbreviations**
CPT1A: carnitine palmitoyltransferase 1A
CPT2: carnitine palmitoyltransferase 2
FAO: fatty acid oxidation
GLS: glutaminase
HRD1: HMG‐CoA reductase degradation protein 1
LC‐MS: liquid chromatography mass spectrometry
TNBC: triple‐negative breast cancer

## 1 Introduction

Triple‐negative breast cancer (TNBC) is a heterogeneous subtype of breast cancer characterized by lack of expression of estrogen receptor (ER) and progesterone receptor (PR) and loss of human epidermal growth factor receptor 2 (HER2) gene amplification. Patients with TNBC have a higher risk of local recurrence or distant metastasis and a poorer outcome than other cancer subtypes [1, 2]. In addition, on account of the lack of response to endocrine therapy‐ and HER2‐targeted therapy, cytotoxic therapy is currently recognized as the mainstay systematic treatment of TNBC. Unfortunately, due to heterogeneity, TNBC is prone to chemotherapy resistance. Therefore, the discovery of new therapeutic targets and predictive markers is extremely urgent for TNBC.

Metabolic reprogramming is an important feature of tumors that is often accompanied by a rapid increase in glucose and glutamine intake [3, 4]. In this regard, TNBC shows a unique glutamine‐dependent phenotype, which means that glutamine supplementation tricarboxylic acid cycle is essential for biomolecule synthesis, energy generation, and glutathione production in TNBC [5, 6]. With the rapid growth of tumors, accelerated glutamine catabolism depletes the local glutamine supply, resulting in glutamine deficiency. Studies have confirmed that glutamine is one of the most depleted metabolites in tumors. However, local glutamine depletion does not inhibit the development of cancer. One of the reasons is that the use of alternative energy sources, such as fatty acids, enables tumor cells to continue to survive and proliferate rapidly in a nutrient‐deficient microenvironment [7, 8, 9]. Recently, dysregulated fatty acid oxidation (FAO) has been linked to TNBC progression. FAO is a multistep process in which fatty acids are acylated to form acyl‐CoA and then enter the mitochondria through carnitine palmityl transferase (CPT). MYC‐overexpressing TNBC cells show an upregulation of CPT activity and an increased dependence on FAO for energy [10, 11]. Increased levels of FAO also activate the oncoprotein Src via autophosphorylation. In addition, inhibition of FAO or knockdown of CPT1 and CPT2 abolishes Src activation and leads to decreased tumor growth and metastasis in TNBC [12]. Of note, metastatic TNBC cells also show an energy dependence on mitochondrial FAO. Despite considerable studies, it remains largely unclear how FAO is elevated in TNBC and, in particular, whether glutamine deficiency is one of the driving factors of the upregulation of FAO.

HMG‐CoA reductase degradation protein 1 (HRD1) was identified as an E3 ligase that controls cholesterol production by regulating the turnover of the rate‐limiting enzyme HMG‐COA reductase (HMGCR) in yeast [13, 14]. Subsequent studies have shown that HRD1 is involved in endoplasmic reticulum‐related degradation (ERAD) and participates in ERAD‐mediated IRE1α ubiquitination degradation [15, 16]. However, with the discovery of a series of new substrate proteins, such as PGC‐1β [17], Nrf2 [18], eIF2α [19], and BLIMP‐1 [20], HRD1 has been proven to regulate cell processes independent of ERAD. In recent years, numerous studies have found that HRD1 plays important roles in lipid metabolism [21, 22]. HRD1 KO in the liver protects mice against obesity, hyperlipidemia, NAFLD, and insulin resistance induced by a high‐fat diet (HFD). HRD1 also participates in the ubiquitylation of VAMP3 to prevent long chain fatty acid (LCFA) uptake [23]. Additionally, HRD1 participates in the regulation of low‐density lipoprotein by binding and promoting the ubiquitin degradation of LOX‐1 [24].

In the current study, we demonstrated that HRD1 was downregulated in TNBC tissues and dysregulated FAO by ubiquitinating CPT2, especially under the condition of glutamine deficiency. In addition, we also found that the curative effect of CB839, a glutaminase (GLS) inhibitor, is more prominent in TNBC cells and tumors with high HRD1 expression. These findings provide insight into HRD1 as a regulator of lipid metabolism and have important implications for TNBC therapeutic targeting.

## 2 Materials and methods

### 2.1 Clinical specimens

Human breast cancer tissue microarrays were obtained from Shanghai Outdo Biotech Company (Shanghai, China). Ten pairs of TNBC samples were obtained from the first affiliated hospital of Dalian Medical University (Dalian, China), and all samples were collected with the informed consent of the patients. The experiments were approved by Research ethics committee at Shanghai Outdo Biotech Companyand the first affiliated hospital of Dalian Medical University. All human samples included in the study were handled in accordance with the tenets of the Declaration of Helsinki.

### 2.2 Antibodies

Anti‐HRD1 (ab118483) and anti‐CPT2 (ab181114) were purchased from Abcam (Cambridge, UK). Ubiquitin antibody (#3933S) and K48‐linkage‐specific polyubiquitin (#8081S) were purchased from Cell Signaling Technology (CST, Danvers, MA, USA). Anti‐Flag tag (20543‐1‐AP), Myc tag (60003‐2‐Ig), anti‐Ki‐67 (27309‐1‐AP), and anti‐CPT1A (15184‐1‐AP) were purchased from ProteinTech (ProteinTech Group, Inc., Rosemont, IL, USA). Anti‐Vinculin (sc‐73614) and anti‐HA tag (sc‐7392) were purchased from Santa Cruz Biotechnology (Santa Cruz, CA, USA).

### 2.3 Inhibitors

The proteasome inhibitor MG132 (S2619) was purchased from Selleckchem (Houston, TX, USA). CB839 (HY‐114334) was purchased from MedChem Express (Monmouth Junction, NJ, USA). The protein synthesis inhibitor cycloheximide (CHX; #2112S) was purchased from Cell Signaling Technology (CST).

### 2.4 Cell culture

MDA‐MB‐231 cells and HEK 293T cells were maintained in Dulbecco's modified Eagle's medium (DMEM; GIBCO, Carlsbad, CA, USA) supplemented with 10% FBS and cultivated in a humidified incubator containing 5% CO_2_ at 37 °C. The cell lines were obtained from the cell bank of the Committee on Type Culture Collection of the Chinese Academy of Sciences (Shanghai, China).

### 2.5 Plasmids

HRD1 and CPT2 were subcloned into Flag‐tagged or Myc‐tagged expression vectors and pBoBi expression vectors. Transfection was performed with Lipofectamine 2000 (Invitrogen, Carlsbad, CA, USA).

### 2.6 Lentiviral shRNA cloning and subcell line generation

Lentiviral shRNAs targeting the genes of HRD1 and CPT2 were cloned in pLKO.1 within the AgeI/EcoRI sites at the 3′ end of the human U6 promoter. The targeted sequences were as follows:

HRD1‐sh1 5′‐ACTGCAGTGCCGAACAGTATT‐3′, HRD1‐sh2 5′‐TGATGGGCAAGGTGTTCTTTG‐3′, CPT2‐shRNA 5′‐CTCCGTTGTTCTGAACTTTAA‐3′. The cell lines were transduced with lentiviral particles from the pLKO.Puro shHRD1 or pLKO.BSD shCPT2 vectors. The stable cell lines were maintained with 2 μg·mL^−1^ puromycin (InvivoGen, Pak Shek Kok, Hong Kong, China) (shHRD1) and 8 μg·mL^−1^ blasticidin (InvivoGen) (shCPT2).

### 2.7 Cell Counting Kit‐8 assay

The cells were plated at a density of 3000 cells/well in 96‐well plates in DMEM (11965092; Gibco), glucose‐free DMEM (11966025), and glutamine‐free DMEM medium (10313021). Cell viability was determined by Cell Counting Kit‐8 (CCK‐8) assay (HY‐K0301; MedChemExpress) following the instructions.

### 2.8 Plate colony formation assay and soft agar assay

For the plate colony formation assay, cells were seeded at a density of 500 cells/well in 12‐well plates in complete DMEM. After 24 h, the medium was changed to complete DMEM, glucose‐free DMEM, and glutamine‐free DMEM. The glucose‐free and complete DMEM was changed every 3 days. The glutamine‐free medium was changed every day. After 14 days, the cells were fixed and stained with crystal violet.

For the soft agar assay, 1 × 10^4^ cells were cultured in an upper layer of 0.40% low melting point agarose, followed by a sublayer of 0.7% low melting point agarose. Cells were maintained for 28 days, and 0.5 mL of fresh culture medium was added every 3 days. The colonies were stained with nitrotetrazolium (1 mg·mL^−1^) blue chloride.

### 2.9 Western blot

Western blot analysis was performed with standard methods. Briefly, cells were lysed in radioimmunoprecipitation assay (RIPA) buffer containing protease inhibitors (Sigma, St. Louis, MO, USA) and phosphatase inhibitors (Roche, Sigma, St. Louis MO, USA). Proteins were separated by SDS/PAGE and blotted onto a PVDF membrane (Bio‐Rad, Hercules, CA, USA). Membranes were probed with specific primary antibodies and then with peroxidase‐conjugated secondary antibodies. Equal protein‐sample loading was monitored using Vinculin antibody. The bands were visualized by chemiluminescence Optimax X‐ray Film Processor (PROTEC GmbH & Co.KG, Oberstenfeld, Germany).

### 2.10 Coimmunoprecipitation and Flag bead pulldown assay

Briefly, cells were lysed in NETN lysis buffer. Antibodies against HRD1 and CPT2 were used for immunoprecipitation. For the Flag bead pulldown experiment, HEK 293T cells were transfected with Flag‐tagged protein and lysed in NETN buffer for 20 min at 4 °C. Crude lysates were subjected to centrifugation at 14 500 ***g*** for 15 min at 4 °C. Supernatants were incubated with Anti‐Flag Affinity Gel (Bimake, B23102, Houston, TX, USA) for 4 h. The agaroses were washed three times with NETN buffer. Proteins were eluted by boiling in 1× SDS running buffer and subjected to SDS/PAGE for immunoblotting.

### 2.11 *In vivo* ubiquitination assay

Cells were lysed with ubiquitination buffer containing 1% SDS and boiled immediately at 95 °C for 10 min. The denatured cell lysates were diluted with SDS‐negative RIPA buffer to reduce SDS to 0.2% and then subjected to Co‐IP followed by western blotting with anti‐ubiquitin‐K48, anti‐HA, anti‐MYC‐tagged, or Flag‐tagged antibodies.

### 2.12 Immunohistochemistry

Standard immunohistochemical (IHC) staining procedures were performed according to the instructions of the IHC Kit (KIHC‐5; ProteinTech, Wuhan, China). HRD1 antibody (ab118483 1 : 100; Abcam), CPT2 antibody (ab181114 1 : 100; Abcam), and Ki‐67 antibody (27309‐1‐AP 1 : 20000; ProteinTech) were used as the primary antibodies. EDTA and citrate solution were used for antigen retrieval depending on the antibody instructions. The H‐score was used to assess the staining intensity.

### 2.13 RNA isolation and quantitative real‐time PCR

RNA was isolated from tumor cells using TRIzol reagent. Reverse transcription PCR was performed using the Revert Aid First Strand cDNA synthesis kit (Fermentas; Thermo Fisher Scientific, Inc., Waltham, MA, USA) according to the protocol. Quantitative real‐time PCR was performed using StepOnePlus and the DNA double‐strand‐specific reagent SYBR Green I for detection (Roche Applied Science, Penzberg, Germany). Fold changes were calculated using the Cq method. The results were normalized to β‐actin levels. The primer sequences were as follows:

HRD1‐F: CTTCACCGTTTTTCGGGATGA
HRD1‐R: CCAGGAGGAACATAAGAGAGACA
CPT2‐F: GGGGCCTACCTGGTCAATG
CPT2‐R: TGGGTAAACGAGTTGAGTTGAAA

### 2.14 LC‐MS‐based metabolomic analyses

The experiments were performed as described previously. Briefly, cell culture plates were washed with PBS and snap‐frozen in liquid nitrogen and stored at −80 °C. Then, 1 mL of −20 °C precooled 80% methanol with an internal standard mixture of carnitine C2:0‐d3, carnitine C10:‐d3, FA 16:0‐d3, and free fatty acid (FFA) 18:0‐d3 was added to the culture plate, and cells were scraped off the bottom into an EP tube. After vortexing and centrifugation, the supernatant was pipetted for drying into a CentriVap Centrifugal Vacuum Concentrator (Labconco Corp., Kansas City, MO, USA). The dried residues were stored at −80 °C until analysis. The samples were analyzed with a UPLC (Waters Corp., Milford, MA, USA) coupled to a Triple Q Exactive Mass Spectrometer (Thermo Scientific). A Waters BEH C8 column (100 mm × 2.1 mm, 1.7 μm) was used for separation. The mobile phases were acetonitrile/H2O (60 : 40, v/v) for phase A and isopropanol/acetonitrile (90 : 10, v/v) for phase B, both containing 10 mm ammonium acetate. The flow rate was 0.26 mL·min^−1^. The column temperature was 55 °C. The gradient started with 32% B and was maintained for 1.5 min, then increased to 85% B within 14 min, reaching 97% at 15.6 min. After maintaining for 2.4 min, it returned to the initial 32% B.

The MS capillary temperature was 320℃ with the auxiliary air heating temperature set at 350℃. The sheath gas and auxiliary gas flow rates were set as 45 and 10 units, respectively. The full scan resolution was set as 120 K, the negative mode was used, the *m/z* scan range was 70–1100 Dalton, and the spray voltage was 3 kV.

### 2.15 *In vivo* tumorigenesis study

All work performed with animals was approved by the Institutional Animal Care and Use Committee of Dalian Medical University. Pathogen‐free female athymic nude mice (4–5 weeks old; 18–22 g) were purchased from the Beijing Vital River Laboratory Animal Technology Co., Ltd. (Beijing, China). All mice were housed in specific pathogen‐free (SPF) environments at the Institute of Genome Engineered Animal Models for Human Disease of Dalian Medical University. A total of 1 × 10^7^ cells were injected subcutaneously into nude mice. The tumor volumes were measured using a caliper every other day. After 5 days, the transplanted mice were treated with CB839 via subcutaneous injection (1 mg·mL^−1^; 0.2 mL) or vehicle on a daily basis for 14 consecutive days. The drugs were composed of 5% ethanol, 5% Tween 20, 10% polyethylene glycol (PEG) 400, and 3% F68 in PBS [25]. At 21 days, all mice were euthanized, and tumors were isolated.

### 2.16 Statistical analysis

The Pearson correlation coefficient was used to evaluate the relationship between HRD1 and CPT2 protein expression levels in human TNBC tissues. ANOVA *post hoc* pairwise comparison analysis was used to compare the means from three groups. Student's *t*‐test (unpaired, two‐tailed) was used to compare the differences between two groups. All of the relative protein expression was normalized by imagej (version no.: 1.8.0_112; https://imagej.nih.gov/ij/). Statistical analysis was performed using the spss 18.0 software package (SPSS, Inc., Chicago, IL, USA).

## 3 Results

### 3.1 HRD1 knockdown accelerates TNBC tumor growth *in vivo*

**Fig. 1:**
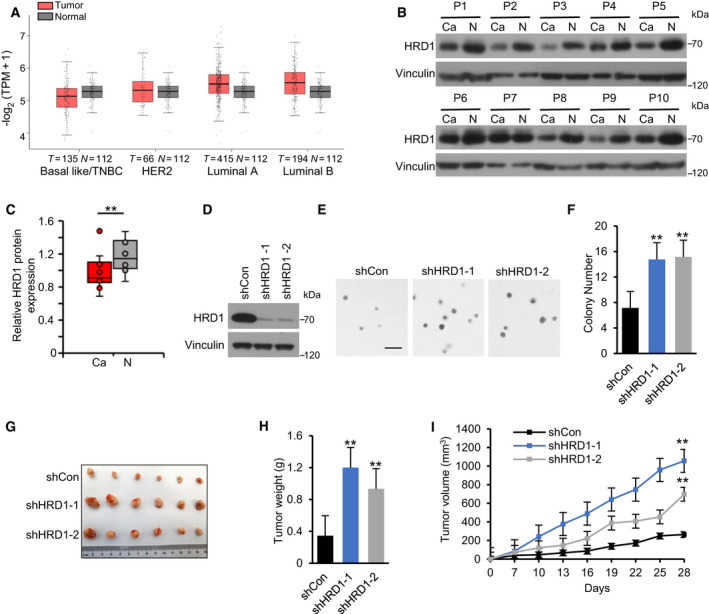
HRD1 knockdown accelerates TNBC tumor growth *in vivo*. (A). HRD1 mRNA in different types of breast cancer (HER2+, Luminal A, Luminal B and TNBC) and normal tissues was analyzed based on TCGA database. The red box represents tumor, and the gray one is normal tissue. http://gepia2.cancer‐pku.cn/#index. (B, C) HRD1 protein expression in 10 pairs of matched adjacent nontumor (NT) and TNBC (Ca) tissues was detected by western blot, and the distributions of HRD1 expressions in both NT and Ca samples were represented by boxplots where expression value was normalized by imagej. **P < 0.01, paired two‐sample Wilcoxon test (*n* = 10 per group). (D) Construction of HRD1‐knockdown stable TNBC cell lines in MDA‐MB‐231 cells. HRD1 proteins were analyzed in stable cells by western blot. (E, F) Cells were cultured in 6‐well plates in an upper layer of 0.40% low melting point agarose, followed by a sublayer of 0.7% low melting point agarose under normal culture condition for 28 days. Then, the number of colonies was calculated and indicated by the histogram. Scale bar = 200 μm. **P < 0.01, ANOVA test. Data were expressed as mean ± SEM (*n* = 3 per group). (G–I) Cells were injected into the right flank of the null mice. Tumor volumes were measured every 3 days. Tumor images (G), weight (H), and growth curves (I) were obtained at day 28 after dissection. **P < 0.01, ANOVA test. Data were expressed as mean ± SEM (*n* = 6 per group).

To evaluate the expression of HRD1 in TNBC, we first analyzed HRD1 mRNA expression in four different types of breast cancer in The Cancer Genome Atlas (TCGA) database and observed that HRD1 mRNA expression was lower in TNBC than in normal breast tissues. In addition, HRD1 mRNA expression in TNBC was also lower than that in the other three types of breast cancer (Fig. 1A). Subsequently, HRD1 protein expression was determined in 10 paired TNBC and adjacent normal fresh tissues. The results showed that HRD1 protein expression was significantly downregulated in TNBC tissues (Fig. 1B,C). Next, we knocked down HRD1 in MDA‐MB‐231 cells with lentiviral small hairpin RNA (shRNA) and constructed HRD1 stable knockdown cell lines (Fig. 1D). A soft agar growth assay showed that HRD1 knockdown promoted the anchorage‐independent growth of MDA‐MB‐231 cells *in vitro* (Fig. 1E,F). Then, subcutaneous xenograft models were applied to investigate the effect of HRD1 on tumorigenesis *in vivo* (Fig. 1G). The tumor volumes and sizes were obviously larger in the HRD1 knockdown groups compared with the control groups (Fig. 1H,I), which indicated that HRD1 knockdown significantly increases xenograft tumor burden. Consistently, these results corroborated an essential role of HRD1 in TNBC tumorigenesis.

### 3.2 HRD1 knockdown promotes the proliferation of TNBC cells *in vitro* under glutamine‐deficient conditions

**Fig. 2:**
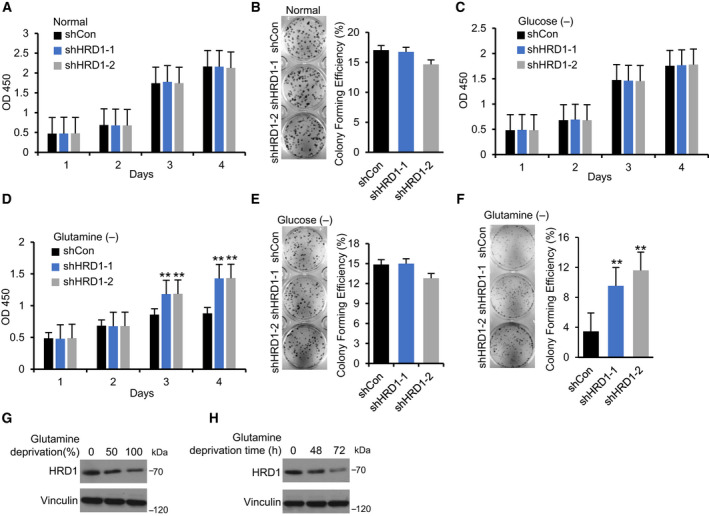
HRD1 knockdown promotes the proliferation of TNBC cells *in vitro* under glutamine‐deficient conditions. (A) Cells were cultured in 96‐well plates under normal culture condition, and cell viability was determined by CCK8 assay. ANOVA test. (B) Cells were cultured in 6‐well plates under normal culture condition for 14 days. Then, the number of colonies was calculated and indicated by the histogram. ANOVA test. (C, E) Cells were cultured in 96‐well plates under glucose‐deficient and glutamine‐deficient condition, respectively, and cell viability was determined by CCK8 assay. **P < 0.01, ANOVA test. (D, F) Cells were cultured in 6‐well plates under glucose‐deficient and glutamine‐deficient condition for 14 days, respectively. Then, the number of colonies was calculated and indicated by the histogram. **P < 0.01, ANOVA test. (G, H) HRD1 protein expression under glutamine deficiency condition in both time‐ and concentration‐dependent cases was analyzed by western blot. Data were expressed as mean ± SEM (*n* = 3 per group).

To further determine the critical role of HRD1 in TNBC, CCK‐8 and plate colony formation assays were performed, and surprisingly, HRD1 knockdown did not promote cell proliferation and colony formation (Fig. 2A,B). The results were inconsistent with the results of the soft agar cloning (Fig. 1E) and *in vivo* experiments (Fig. 1G), and we hypothesized that this discrepancy might be related to the lack of nutrients in the *in vivo* environment. Subsequently, we detected the effect of HRD1 knockdown on cell proliferation in glucose‐ and glutamine‐deficient conditions. Interestingly, HRD1 knockdown did not promote cell proliferation or plate colony formation in a glucose‐deficient environment (Fig. 2C,D) but did promote these behaviors in a glutamine‐deficient environment (Fig. 2E,F). Of note, glutamine deprivation inhibited the expression of HRD1 in both time‐ and concentration‐dependent manners (Fig. 2G,H). This means glutamine deficiency inhibits the expression of HRD1, but this reduction does not overcome the inhibitory effect of glutamine deficiency. Taken together, these data suggest that HRD1 silencing promotes the proliferation of TNBC cells *in vitro* especially under the glutamine‐deficient condition.

### 3.3 HRD1 knockdown upregulates FAO in TNBC cells

**Fig. 3:**
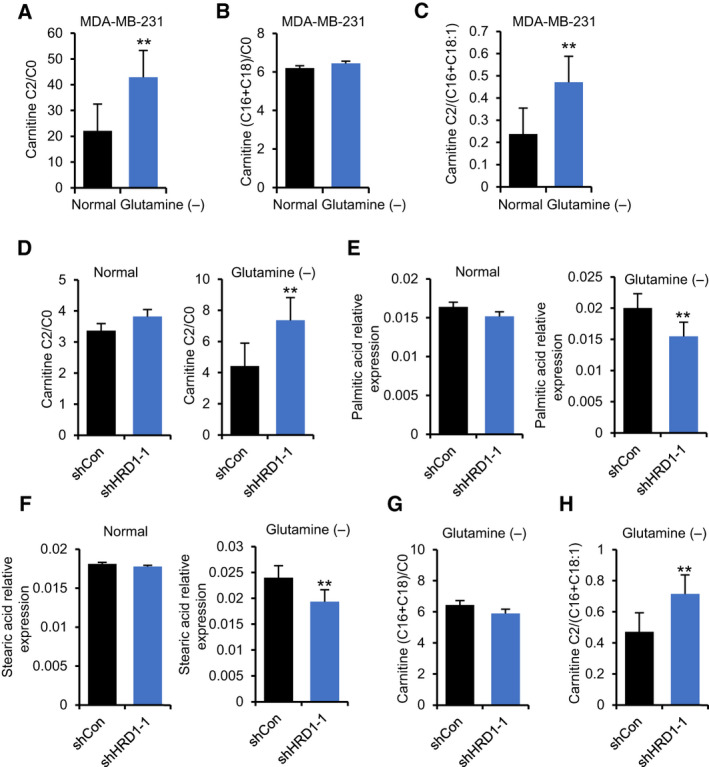
HRD1 knockdown upregulates FAO in TNBC cells. (A–C) Carnitine, carnitine C2:0, carnitine C16:0, carnitine C18:0, and carnitine C18:1 in MDA‐MB‐231 cells under normal culture condition and glutamine deficiency condition were detected by LC‐MS. Then, ratio of carnitine C2/C0, carnitine C2/(C16+C18:1), and carnitine (C16+C18)/C0 was calculated and indicated by the histogram. **P < 0.01, unpaired two‐tailed Student's *t*‐test. (D) Carnitine and carnitine C2:0 in HRD1 knockdown and shCon cells under normal culture condition and glutamine deficiency condition, respectively, were detected by LC‐MS. Then, ratio of carnitine C2/C0 was calculated and indicated by the histogram. **P < 0.01, unpaired two‐tailed Student's *t*‐test. (E, F) Palmitic acid and Stearic acid in HRD1 knockdown and shCon cells under normal culture condition and glutamine deficiency condition, respectively, were detected by LC‐MS. **P < 0.01, unpaired two‐tailed Student's *t*‐test. (G) Carnitine, carnitine C16:0, carnitine C18:0, and carnitine C18:1 in HRD1 knockdown and shCon cells under glutamine deficiency condition were detected by LC‐MS. Then, ratio of carnitine C2/(C16+C18:1) and carnitine (C16+C18)/C0 was calculated and indicated by the histogram. **P < 0.01, unpaired two‐tailed Student's *t*‐test. Data were expressed as mean ± SEM (*n* = 5 per group).

To determine whether fatty acids were utilized as an alternative energy source after glutamine deprivation, LC‐MS was used to detect the contents of carnitine, palmitic acid, and stearic acid. As expected, the ratio of carnitine C2/C0 increased significantly after glutamine deprivation (Fig. 3A), which represented the acceleration of FAO. In addition, we also found that the ratio of carnitine C2/(C16+C18:1) was upregulated (Fig. 3C), while carnitine (C16+C18)/C0 was not changed (Fig. 3B), which indicated that the activity of CPT2 was significantly increased while the activity of CPT1 was not changed after glutamine deprivation. These results suggest that the acceleration of FAO after glutamine deprivation mainly depends on the upregulation of CPT activity.

We next sought to explore whether HRD1 is involved in the utilization of fatty acids after glutamine deprivation. The results showed that under glutamine deprivation conditions, but not under normal conditions, HRD1 knockdown obviously upregulated the ratio of carnitine C2/C0 (Fig. 3D). In addition, after glutamine deprivation, the contents of palmitic acid and stearic acid in HRD1 knockdown cells were also significantly decreased (Fig. 3E,F). Similarly, there was also no change in the two fatty acids under normal culture conditions (Fig. 3E,F). Furthermore, we observed that HRD1 knockdown significantly upregulated the ratio of carnitine C2/(C16+C18:1) but not carnitine (C16+C18)/C0 (Fig. 3G,H), which indicated that the activity of CPT2 was significantly increased while CPT1 was not changed after HRD1 knockdown under glutamine deprivation conditions. These results collectively suggest that HRD1 knockdown significantly promotes FAO in the case of glutamine deficiency mainly via the upregulation of CPT2 activity.

### 3.4 HRD1 interacts with CPT2 and decreases its protein stability

**Fig. 4:**
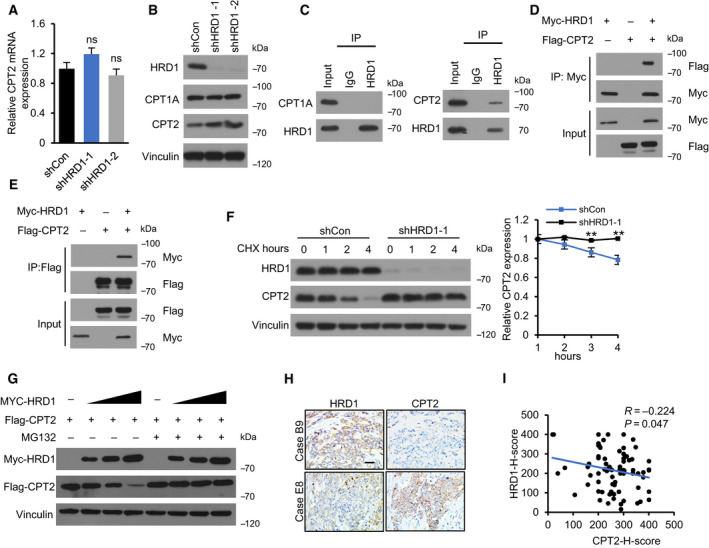
HRD1 interacts with CPT2 and decreases its protein stability. (A) CPT2 mRNA was detected in HRD1 knockdown cells by qRT–PCR. *P < 0.05, **P < 0.01, ns = not significant, ANOVA test. Data were expressed as mean ± SEM (*n* = 3 per group). (B) CPT1A and CPT2 protein expression was detected in HRD1 knockdown cells by western blot. (C) Cell lysates of MDA‐MB‐231 cells were immunoprecipitated with an IgG or HRD1 antibodies, and immunoblot assays were performed using HRD1, CPT2, or CPT1A antibody. (D, E) HEK 293T cells were transfected for 24 h with plasmids encoding either Flag‐CPT2 or Myc‐HRD1 alone or in combination. Cell lysates were immunoprecipitated with an anti‐Flag and anti‐Myc antibodies, and immunoblot assays were performed using anti‐Myc or anti‐Flag antibody. (F) HRD1 knockdown and shCon cells were treated with 40 μm cycloheximide for the indicated times. Cell lysates were immunoblotted with HRD1 and CPT2 antibodies. Relative levels of CPT2 were quantified by imagej. *P < 0.05, **P < 0.01, unpaired two‐tailed Student's *t*‐test. Data were expressed as mean ± SEM (*n* = 3 per group). (G) HEK 293T cells were transfected with Flag‐CPT2 alone or together with increasing doses (0.5, 1.0, and 1.5 μg) of Myc‐HRD1 for 24 h, and treated with or without 10 μm MG132 for 6 h. The expression of transfected CPT2 and HRD1 was confirmed by immunoblotting with anti‐Flag and anti‐Myc antibody. (H) Representative IHC staining of HRD1 and CPT2 in TNBC tissues. Scale bars, 50 μm. (I) Correlation analysis between HRD1 and CPT2 based on H‐Score. R represents Pearson correlation coefficient.

Next, we determined whether HRD1 affects intracellular CPT2 levels. We observed that HRD1 knockdown significantly upregulated CPT2 protein expression (Fig. 4B) but not CPT2 mRNA expression (Fig. 4A). Notably, HRD1 knockdown did not affect CPT1A protein expression (Fig. 4B). Then, coimmunoprecipitation using cell lysates validated the specific interaction between endogenous HRD1 and CPT2, but not CPT1, in MDA‐MB‐231 cells (Fig. 4C). To validate the physical interaction, we enforced the expression of Myc‐HRD1 and Flag‐CPT2 in HEK 293T cells for reciprocal immunoprecipitation and confirmed the interaction between these two proteins (Fig. 4D,E). We next sought to explore whether HRD1 decreases CPT2 protein stability. Half‐life measurements using cycloheximide revealed that HRD1 knockdown increased the half‐life of CPT2 protein (Fig. 4F). Moreover, HRD1 transfection plus MG132 treatment did not substantially alter the CPT2 levels. Further, in cells without MG132 treatment, the decrease in CPT2 protein expression in response to HRD1 transfection was dose‐dependent (Fig. 4G). To further confirm the role of HRD1 in the regulation of CPT2 in TNBC, we detected the expression of HRD1 and CPT2 in a TNBC tissue microarray by IHC (Fig. 4H). The results showed that HRD1 protein was notably negatively related to CPT2 protein (Fig. 4I). Taken together, these data suggest that HRD1 selectively and specifically downregulates intracellular CPT2 levels.

### 3.5 HRD1 ubiquitinates CPT2 by targeting K48 ubiquitination

**Fig. 5:**
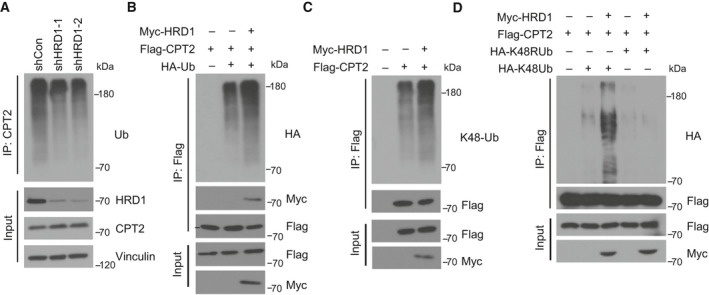
HRD1 ubiquitinates CPT2 by targeting K48 ubiquitination. (A) Immunoprecipitation of cell lysates of HRD1 knockdown and shCon cells was performed with CPT2 antibody, and then, lysates were immunoblotted with Ub, HRD1, CPT2, and Vinculin antibody. (B) HEK 293T cells were transfected for 24 h with HA‐Ub, Flag‐CPT2, and Myc‐HRD1 alone or in combination, and treated for 6 h with 10 μm MG132. Immunoprecipitation of cell lysates was performed with Anti‐Flag Affinity Gel, and lysates were immunoblotted with HA, Flag, and Myc antibody. (C) HEK 293T cells were transfected for 24 h with Flag‐CPT2 and Myc‐HRD1 alone or in combination, and treated for 6 h with 10 μm MG132. Immunoprecipitation of cell lysates was performed with Anti‐Flag Affinity Gel, and lysates were immunoblotted with K48‐Ub, Flag, and Myc antibody. (D) HEK 293T cells were transfected for 24 h with HA‐K48Ub, HA‐K48RUb, Flag‐CPT2, and Myc‐HRD1 alone or in combination, and treated for 6 h with 10 μm MG132. Immunoprecipitation of cell lysates was performed with Anti‐Flag Affinity Gel, and lysates were immunoblotted with HA, Flag, and Myc antibody.

To determine the underlying mechanism of HRD1‐mediated negative regulation of CPT2 levels, we tested whether HRD1 affected CPT2 ubiquitination. First, HRD1 knockdown significantly inhibited HRD1 ubiquitination in MDA‐MB‐231 cells (Fig. 5A). Next, we cotransfected different combinations of Flag‐CPT2, Myc‐HRD1, and HA‐tagged wild‐type ubiquitin and found that HRD1 ubiquitination was significantly increased when HRD1 was introduced (Fig. 5B). K48‐linked ubiquitin chains are generally known to label proteins for proteasomal degradation. Therefore, we examined whether HRD1‐mediated HRD1 ubiquitination specifically targeted K48 chains. We also cotransfected different combinations of Flag‐CPT2 and Myc‐HRD1 plasmids, and then, cell lysates were immunoprecipitated with an anti‐Flag antibody. Immunoblot analysis with anti‐K48 ubiquitin antibodies was performed. As expected, HRD1 significantly upregulated K48 ubiquitin chain‐mediated CPT2 ubiquitination (Fig. 5C). To further verify these results, we added HA‐K48 or HA‐K48R ubiquitin chains into the cotransfection system and found that CPT2 was specifically ubiquitinated through K48‐specific chains when HRD1 was introduced (Fig. 5D). These results demonstrate that HRD1 ubiquitinates CPT2, which is processed through Lys‐48‐linked ubiquitin chains.

### 3.6 HRD1 knockdown promotes TNBC cell proliferation and tumorigenesis in a CPT2‐dependent manner

**Fig. 6:**
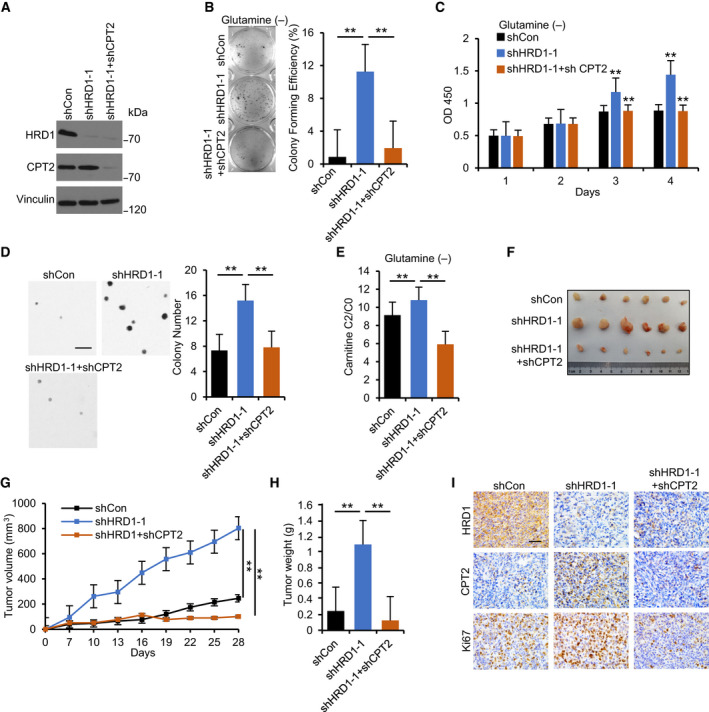
HRD1 knockdown promotes TNBC cell proliferation and tumorigenesis in a CPT2‐dependent manner. (A) Construction of MDA‐MB‐231‐shHRD1+shCPT2 stable cell line. HRD1 and CPT2 proteins were analyzed in stable cells by western blot. (B) Cells were cultured in 6‐well plates under glutamine‐deficient condition for 14 days. Then, the number of colonies was calculated and indicated by the histogram. **P < 0.01, ANOVA test. Data were expressed as mean ± SEM (*n* = 3 per group). (C) Cells were cultured in 96‐well plates under glutamine‐deficient condition, and cell viability was determined by CCK8 assay. **P < 0.01, ANOVA Test. Data were expressed as mean ± SEM (*n* = 3 per group). (D) Cells were cultured in 6‐well plates in an upper layer of 0.40% low melting point agarose, followed by a sublayer of 0.7% low melting point agarose under normal culture condition for 28 days. Scale bar = 200 μm. Then, the number of colonies was calculated and indicated by the histogram. **P < 0.01, ANOVA test. Data were expressed as mean ± SEM (*n* = 3 per group). (E) Carnitine and carnitine C2:0 in stable cells under glutamine deficiency condition were detected by LC‐MS. Then, ratio of carnitine C2/C0 was calculated and indicated by the histogram. **P < 0.01, unpaired two‐tailed Student's *t*‐test. Data were expressed as mean ± SEM (*n* = 5 per group). (F–H) Cells were injected into the right flank of the null mice. Tumor volumes were measured every 3 days. Tumor images (F), growth curves (G), and weight (H) were obtained at day 28 after dissection. **P < 0.01, ANOVA test. Data were expressed as mean ± SEM (*n* = 6 per group). (I) Representative IHC staining of HRD1, CPT2, and Ki67 in xenograft tissues. Scale bars, 50 μm.

To further clarify whether HRD1 is involved in the regulation of cell proliferation through CPT2, we knocked down CPT2 in HRD1 stable knockdown MDA‐MB‐231 cells (Fig. 6A). Plate clone formation assays and proliferation experiments confirmed that CPT2 knockdown counteracted the HRD1 knockdown‐mediated upregulation of proliferation and clone formation rate in the presence of glutamine deficiency (Fig. 6B,C). Meanwhile, the ratio of carnitine C2/C0 also changed in the same trend (Fig. 6E). Soft agar colony formation (Fig. 6D) and *in vivo* tumorigenesis experiments (Fig. 6F–H) further proved that knockdown of CPT2 inhibited HRD1 knockdown‐promoted tumorigenesis. In addition, IHC staining of tumor sections showed that CPT2 knockdown markedly reduced proliferation as indicated by Ki‐67 staining (Fig. 6I). These results suggest that CPT2 plays a key role in HRD1‐mediated TNBC tumorigenesis.

### 3.7 HRD1 is a potential predictor of the efficacy of GLS inhibitors

**Fig. 7:**
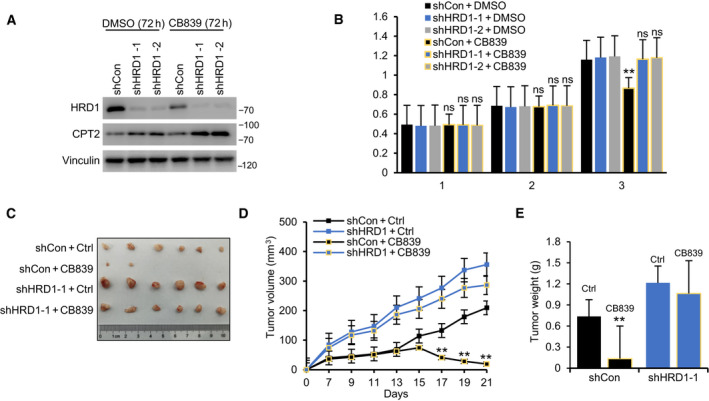
HRD1 is a potential predictor of the efficacy of GLS inhibitors. (A) Stable cells were treated with 10 μmol CB839 or DMSO for 72 h, respectively. HRD1 and CPT2 proteins were analyzed in stable cells by western blot. (B) Stable cells were cultured in 96‐well plates under normal culture condition, and treated with CB839 and DMSO, respectively. Cell viability was determined by CCK8 assay. **P < 0.01, ns = not significant, ANOVA test. Data were expressed as mean ± SEM (*n* = 3 per group). (C–E) Stable cells were injected into the right flank of the null mice. After 5 days, the transplanted mice were treated with CB839 via subcutaneous injection (1 mg·mL^−1^; 0.2 mL) or vehicle on a daily basis for 14 consecutive days. Tumor volumes were measured every other day. Tumor images (B), growth curves (C), and weight (D) were obtained at day 21 after dissection. **P < 0.01, ANOVA test. Data were expressed as mean ± SEM (*n* = 6 per group).

Glutaminase is a restriction enzyme in the process of glutamine catabolism. Previous studies have confirmed that the FAO level is markedly upregulated in CB839 (a GLS inhibitor, which is undergoing phase I‐II clinical trials in TNBC)‐resistant TNBC cell lines, and CB839 combined with FAO inhibition is a feasible method for combating CB839 resistance in TNBC. Our previous experiments confirmed that HRD1 is a key node in the upregulation of FAO under glutamine‐deficient conditions in TNBC. We speculate that HRD1 may be a potential predictor of the efficacy of GLS inhibitors. First, we performed immunoblotting analysis of HRD1 and CPT2 after CB839 treatment and found that similar to the results of glutamine deficiency, CB839 treatment also inhibited the expression of HRD1. In addition, under CB839 treatment, we also observed that HRD1 knockdown could significantly upregulate the expression of CPT2 (Fig. 7A). Next, we found that CB839 significantly inhibited cell proliferation in the control group rather than HRD1 knockdown group (Fig. 7B). Subsequent *in vivo* experiments confirmed that CB839 treatment significantly inhibited tumorigenesis in cells with high HRD1 expression (Fig. 7C–E). Overall, combined with previous findings, these data indicate that high expression of HRD1 is a potential predictor of the efficacy of CB839.

## 4 Discussion

Due to the characteristics of unlimited proliferation, tumor cells consume nutrients such as glucose and glutamine significantly faster than normal cells. At the same time, due to the abnormal growth of tumor blood vessels, the supply of nutrients is insufficient, which eventually leads to the nutritional deficiency of tumor cells. Studies have found that compared with normal tissues, glutamine in tumor tissue can be reduced to almost undetectable levels, and the glutamine level in the tumor center is significantly lower than that in the surrounding region [26, 27]. However, tumor cells can quickly adapt to the adversity of nutritional deficiencies and maintain rapid growth, which is achieved by changing the metabolic mode of tumor cells, that is, metabolic reprogramming [3, 28]. In this study, we found that HRD1 knockdown upregulated soft agar colony formation ability and tumorigenesis in TNBC cells. Furthermore, we observed that HRD1 knockdown merely promoted cell proliferation and plate colony formation under glutamine deprivation conditions. In addition, HRD1 expression was significantly downregulated after glutamine deprivation. Combined with these results, we speculate that HRD1 may be a key node of metabolic reprogramming in MDA‐MB‐231 cells under glutamine deficiency.

Glutamine addiction and acceleration of FAO are both metabolic characteristics of most TNBC [29]. Previous studies have found that high expression of GLS is associated with high grade and high metastatic potential in breast cancer. GLS knockdown and the application of GLS inhibitors show encouraging antitumor effects in TNBC [30, 31, 32]. FAO promotes the autophosphorylation of Src protein Y419 in TNBC cells. Activated Src further phosphorylates mitochondrial electron transport chain (ETC) proteins, thereby maintaining mitochondrial function and providing sufficient energy for the invasion and metastasis of TNBC [12]. More importantly, the upregulation of FAO is related to the metabolic adaptation of different types of tumors under nutritional deprivation and hypoxia. Studies have shown that glutamine deprivation leads to the upregulation of FAO‐related proteins. Interestingly, the FAO level was significantly upregulated in CB839‐resistant TNBC cell lines, and the application of FAO inhibitors reversed CB839 resistance. Combined with the above results, we speculate that under glutamine deprivation conditions, HRD1 knockdown may promote tumorigenesis by upregulating FAO in TNBC.

To confirm this hypothesis, LC‐MS was used to detect the contents of acylcarnitine and palmitic acid under normal culture conditions and glutamine deprivation conditions. By calculating the ratio of acylcarnitine, we found that the FAO level was significantly upregulated after glutamine deprivation. In addition, HRD1 knockdown significantly increased the FAO level and CPT2 activity in glutamine‐deprived cells. These results confirmed our previous hypothesis and suggested that this metabolic alteration may be related to the upregulation of CPT2 activity mediated by HRD1 knockdown.

The conversion of fatty acids to acyl‐COA and the subsequent transport of fatty acyl‐COA to mitochondria through CPT1 and 2 constitute the first step in FAO. CPTs are the key enzymes in this process. Myc‐overexpressing TNBC samples exhibit upregulation of CPT activity and increased dependence on FAO for energy [10, 11]. We speculate that HRD1 may be directly involved in the regulation of CPT1 or CPT2. Indeed, we found that HRD1 knockdown was accompanied by an upregulation of CPT2 protein expression, while there was no significant change in CPT1A. Antibody‐dependent endogenous Co‐IP showed that HRD1 interacts directly with CPT2, but not with CPT1, in MDA‐MB‐231 cells. Protein half‐life and ubiquitin experiments further confirmed that HRD1 promoted the ubiquitin‐mediated degradation of CPT2. To further clarify whether HRD1 affects FAO and tumorigenesis in glutamine deficiency conditions through CPT2, we knocked down the expression of CPT2 by knocking down HRD1. The results showed that knocking down CPT2 significantly inhibited FAO acceleration and tumorigenesis caused by HRD1 knockdown.

The GLS inhibitor CB839 is an effective and selective small molecular inhibitor that is currently in stage 1/2 clinical trials in TNBC. In a xenotransplantation model, it has been proven that CB839 can effectively inhibit the growth of TNBC and other glutamine‐dependent tumors. However, after inhibition of glutamine catabolism, the significant acceleration of FAO in TNBC cells leads to resistance to CB839. The combination of GLS inhibitors with FAO inhibitors is considered to be a feasible way to address the resistance of TNBC to GLS inhibitors [33, 34]. Since previous data have confirmed that HRD1 is a key node of metabolic reprogramming in TNBC cells under glutamine‐deficient conditions, we speculate that HRD1 may be a potential predictor of GLS inhibitor efficacy in TNBC therapy. Subsequent *in vivo* and *in vitro* experiments confirmed that the inhibitory effect of the GLS inhibitor on tumors decreased significantly after HRD1 knockdown. This suggested that high expression of HRD1 may indicate better CB839 efficacy in TNBC treatment.

## 5 Conclusion

In summary, the current study identified and verified a key regulatory protein, HRD1, that regulates FAO and tumorigenesis in TNBC and elucidated the specific mechanism: (a) HRD1 knockdown increases the FAO level of TNBC cells after glutamine deprivation by upregulating CPT2 activity. (b) HRD1 interacts with and mediates the ubiquitin‐mediated degradation of the CPT2 protein. (c) HRD1 participates in FAO and tumorigenesis through CPT2. Additionally, high expression of HRD1 may be a predictor of the therapeutic effect of the GLS inhibitor CB839 in TNBC. These findings provide insight into HRD1 as a regulator of lipid metabolism and have important implications for TNBC therapeutic targeting.

## Conflict of interest

The authors declare no conflict of interest.

## Author contributions

XG, AW, and HW conceived the project, and HP and HW supervised the project. XG, AW, and WW designed and performed most of experiments and the data analysis. YW, HC, XL, TX, AZ, DC, HQ, and TL provided significant intellectual input. XG, HW, AW, and HP wrote the manuscript with input from all other authors.

### Peer Review

The peer review history for this article is available at https://publons.com/publon/10.1002/1878‐0261.12856.

## References

[1] Brenton JD , Carey LA , Ahmed AA & Caldas C (2005) Molecular classification and molecular forecasting of breast cancer: ready for clinical application? J Clin Oncol 23, 7350–7360. 16145060 10.1200/JCO.2005.03.3845

[2] Gluz O , Liedtke C , Gottschalk N , Pusztai L , Nitz U & Harbeck N (2009) Triple‐negative breast cancer–current status and future directions. Ann Oncol 20, 1913–1927. 19901010 10.1093/annonc/mdp492

[3] Pavlova NN & Thompson CB (2016) The emerging hallmarks of cancer metabolism. Cell Metab 23, 27–47. 26771115 10.1016/j.cmet.2015.12.006 PMC4715268

[4] Sun X , Wang M , Wang M , Yu X , Guo J , Sun T , Li X , Yao L , Dong H & Xu Y (2020) Metabolic reprogramming in triple‐negative breast cancer. Front Oncol 10, 428. 32296646 10.3389/fonc.2020.00428 PMC7136496

[5] Lampa M , Arlt H , He T , Ospina B , Reeves J , Zhang B , Murtie J , Deng G , Barberis C , Hoffmann D *et al* (2017) Glutaminase is essential for the growth of triple‐negative breast cancer cells with a deregulated glutamine metabolism pathway and its suppression synergizes with mTOR inhibition. PLoS One 12, e0185092. 28950000 10.1371/journal.pone.0185092 PMC5614427

[6] Winnike JH , Stewart DA , Pathmasiri WW , McRitchie SL & Sumner SJ (2018) Stable isotope‐resolved metabolomic differences between hormone‐responsive and triple‐negative breast cancer cell lines. Int J Breast Cancer 2018, 2063540. 30363973 10.1155/2018/2063540 PMC6186330

[7] Halama A , Kulinski M , Dib SS , Zaghlool SB , Siveen KS , Iskandarani A , Zierer J , Prabhu KS , Satheesh NJ , Bhagwat AM *et al* (2018) Accelerated lipid catabolism and autophagy are cancer survival mechanisms under inhibited glutaminolysis. Cancer Lett 430, 133–147. 29777783 10.1016/j.canlet.2018.05.017

[8] Altman BJ , Stine ZE & Dang CV (2016) From Krebs to clinic: glutamine metabolism to cancer therapy. Nat Rev Cancer 16, 619–634. 27492215 10.1038/nrc.2016.71 PMC5484415

[9] Lyssiotis CA & Kimmelman AC (2017) Metabolic interactions in the tumor microenvironment. Trends Cell Biol 27, 863–875. 28734735 10.1016/j.tcb.2017.06.003 PMC5814137

[10] Camarda R , Zhou AY , Kohnz RA , Balakrishnan S , Mahieu C , Anderton B , Eyob H , Kajimura S , Tward A , Krings G *et al* (2016) Inhibition of fatty acid oxidation as a therapy for MYC‐overexpressing triple‐negative breast cancer. Nat Med 22, 427–432. 26950360 10.1038/nm.4055 PMC4892846

[11] Casciano JC , Perry C , Cohen‐Nowak AJ , Miller KD , Vande Voorde J , Zhang Q , Chalmers S , Sandison ME , Liu Q , Hedley A *et al* (2020) MYC regulates fatty acid metabolism through a multigenic program in claudin‐low triple negative breast cancer. Br J Cancer 122, 868–884. 31942031 10.1038/s41416-019-0711-3 PMC7078291

[12] Park JH , Vithayathil S , Kumar S , Sung PL , Dobrolecki LE , Putluri V , Bhat VB , Bhowmik SK , Gupta V , Arora K *et al* (2016) Fatty acid oxidation‐driven Src links mitochondrial energy reprogramming and oncogenic properties in triple‐negative breast cancer. Cell Rep 14, 2154–2165. 26923594 10.1016/j.celrep.2016.02.004 PMC4809061

[13] Hampton R & Rine J (1994) Regulated degradation of HMG‐CoA reductase, an integral membrane protein of the endoplasmic reticulum, in yeast. J Cell Biol 125, 299–312. 8163547 10.1083/jcb.125.2.299 PMC2120026

[14] Hampton R , Gardner R & Rine J (1996) Role of 26S proteasome and HRD genes in the degradation of 3‐hydroxy‐3‐methylglutaryl‐CoA reductase, an integral endoplasmic reticulum membrane protein. Mol Biol Cell 7, 2029–2044. 8970163 10.1091/mbc.7.12.2029 PMC276048

[15] Okuda‐Shimizu Y & Hendershot LM (2007) Characterization of an ERAD pathway for nonglycosylated BiP substrates, which require Herp. Mol Cell 28, 544–554. 18042451 10.1016/j.molcel.2007.09.012 PMC2149893

[16] Christianson JC , Olzmann JA , Shaler TA , Sowa ME , Bennett EJ , Richter CM , Tyler RE , Greenblatt EJ , Harper JW & Kopito RR (2011) Defining human ERAD networks through an integrative mapping strategy. Nat Cell Biol 14, 93–105. 22119785 10.1038/ncb2383 PMC3250479

[17] Fujita H , Yagishita N , Aratani S , Saito‐Fujita T , Morota S , Yamano Y , Hansson MJ , Inazu M , Kokuba H , Sudo K *et al* (2015) The E3 ligase synoviolin controls body weight and mitochondrial biogenesis through negative regulation of PGC‐1beta. EMBO J 34, 1042–1055. 25698262 10.15252/embj.201489897 PMC4406651

[18] Wu T , Zhao F , Gao B , Tan C , Yagishita N , Nakajima T , Wong PK , Chapman E , Fang D & Zhang DD (2014) Hrd1 suppresses Nrf2‐mediated cellular protection during liver cirrhosis. Genes Dev 28, 708–722. 24636985 10.1101/gad.238246.114 PMC4015486

[19] Huang Y , Sun Y , Cao Y , Sun H , Li M , You H , Su D , Li Y & Liang X (2017) HRD1 prevents apoptosis in renal tubular epithelial cells by mediating eIF2alpha ubiquitylation and degradation. Cell Death Dis 8, 3202. 29233968 10.1038/s41419-017-0002-y PMC5870601

[20] Yang H , Qiu Q , Gao B , Kong S , Lin Z & Fang D (2014) Hrd1‐mediated BLIMP‐1 ubiquitination promotes dendritic cell MHCII expression for CD4 T cell priming during inflammation. J Exp Med 211, 2467–2479. 25366967 10.1084/jem.20140283 PMC4235642

[21] Wei J , Yuan Y , Chen L , Xu Y , Zhang Y , Wang Y , Yang Y , Peek CB , Diebold L , Yang Y *et al* (2018) ER‐associated ubiquitin ligase HRD1 programs liver metabolism by targeting multiple metabolic enzymes. Nat Commun 9, 3659. 30201971 10.1038/s41467-018-06091-7 PMC6131148

[22] Jiang W & Song BL (2014) Ubiquitin ligases in cholesterol metabolism. Diabetes Metab J 38, 171–180. 25003069 10.4093/dmj.2014.38.3.171 PMC4083022

[23] Yu M , Du H , Wang B , Chen J , Lu F , Peng S , Sun Y , Liu N , Sun X , Shiyun D *et al* (2020) Exogenous H2S induces Hrd1 S‐sulfhydration and prevents CD36 translocation via VAMP3 ubiquitylation in diabetic hearts. Aging Dis 11, 286–300. 32257542 10.14336/AD.2019.0530 PMC7069459

[24] Li Q , Xuan W , Jia Z , Li H , Li M , Liang X & Su D (2020) HRD1 prevents atherosclerosis‐mediated endothelial cell apoptosis by promoting LOX‐1 degradation. Cell Cycle 19, 1466–1477. 32308114 10.1080/15384101.2020.1754561 PMC7469520

[25] Chen Z , Li D , Xu N , Fang J , Yu Y , Hou W , Ruan H , Zhu P , Ma R , Lu S *et al* (2019) Novel 1,3,4‐selenadiazole‐containing kidney‐type glutaminase inhibitors showed improved cellular uptake and antitumor activity. J Med Chem 62, 589–603. 30543285 10.1021/acs.jmedchem.8b01198

[26] Leone RD , Zhao L , Englert JM , Sun IM , Oh MH , Sun IH , Arwood ML , Bettencourt IA , Patel CH , Wen J *et al* (2019) Glutamine blockade induces divergent metabolic programs to overcome tumor immune evasion. Science 366, 1013–1021. 31699883 10.1126/science.aav2588 PMC7023461

[27] Pan M , Reid MA , Lowman XH , Kulkarni RP , Tran TQ , Liu X , Yang Y , Hernandez‐Davies JE , Rosales KK , Li H *et al* (2016) Regional glutamine deficiency in tumours promotes dedifferentiation through inhibition of histone demethylation. Nat Cell Biol 18, 1090–1101. 27617932 10.1038/ncb3410 PMC5536113

[28] Wang Z , Jiang Q & Dong C (2020) Metabolic reprogramming in triple‐negative breast cancer. Cancer Biol Med 17, 44–59. 32296576 10.20892/j.issn.2095-3941.2019.0210 PMC7142847

[29] Wright H , Hou J , Xu B , Cortez M , Potma E , Tromberg B & Razorenova O (2017) CDCP1 drives triple‐negative breast cancer metastasis through reduction of lipid‐droplet abundance and stimulation of fatty acid oxidation. Proc Natl Acad Sci USA 114, E6556–E6565. 28739932 10.1073/pnas.1703791114 PMC5559020

[30] Bhutia YD , Babu E , Ramachandran S & Ganapathy V (2015) Amino Acid transporters in cancer and their relevance to “glutamine addiction”: novel targets for the design of a new class of anticancer drugs. Cancer Res 75, 1782–1788. 25855379 10.1158/0008-5472.CAN-14-3745

[31] Wise DR & Thompson CB (2010) Glutamine addiction: a new therapeutic target in cancer. Trends Biochem Sci 35, 427–433. 20570523 10.1016/j.tibs.2010.05.003 PMC2917518

[32] Xiang Y , Stine ZE , Xia J , Lu Y , O'Connor RS , Altman BJ , Hsieh AL , Gouw AM , Thomas AG , Gao P *et al* (2015) Targeted inhibition of tumor‐specific glutaminase diminishes cell‐autonomous tumorigenesis. J Clin Invest 125, 2293–2306. 25915584 10.1172/JCI75836 PMC4497742

[33] Reis LMD , Adamoski D , Ornitz Oliveira Souza R , Rodrigues Ascencao CF , Sousa de Oliveira KR , Correa‐da‐Silva F , Malta de Sa Patroni F , Meira Dias M , Consonni SR , Mendes de Moraes‐Vieira PM *et al* (2019) Dual inhibition of glutaminase and carnitine palmitoyltransferase decreases growth and migration of glutaminase inhibition‐resistant triple‐negative breast cancer cells. J Biol Chem 294, 9342–9357. 31040181 10.1074/jbc.RA119.008180 PMC6579458

[34] Qu Q , Zeng F , Liu X , Wang QJ & Deng F (2016) Fatty acid oxidation and carnitine palmitoyltransferase I: emerging therapeutic targets in cancer. Cell Death Dis 7, e2226. 27195673 10.1038/cddis.2016.132 PMC4917665

